# Supplementary material for: Increased O-GlcNAcylation by Upregulation of Mitochondrial O-GlcNAc Transferase (mOGT) Inhibits the Activity of Respiratory Chain Complexes and Controls Cellular Bioenergetics
Source: Cancers (Basel). 2024 Mar 5;16(5):1048. doi: 10.3390/cancers16051048 (PMC10930986; doi:10.3390/cancers16051048)
Supplement: Supplementary file 1 [file cancers-16-01048-s001.zip › cancers-2826233-Western blotting.pdf]

cancers

MDPI

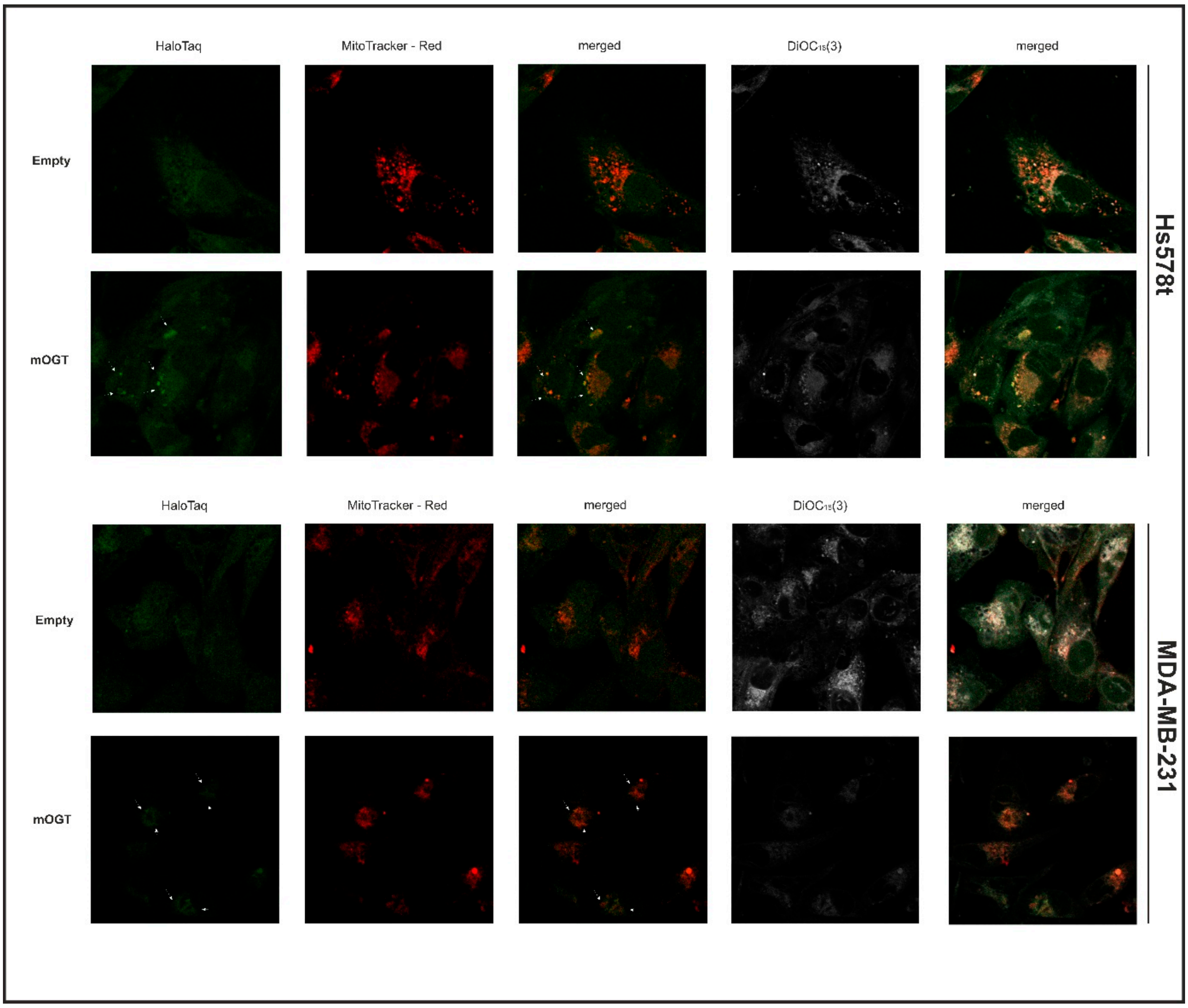

Figure S1. mOGT targets mitochondria in breast cancer MDA-MB-231 and Hs578t cell lines.

Figure 1B.

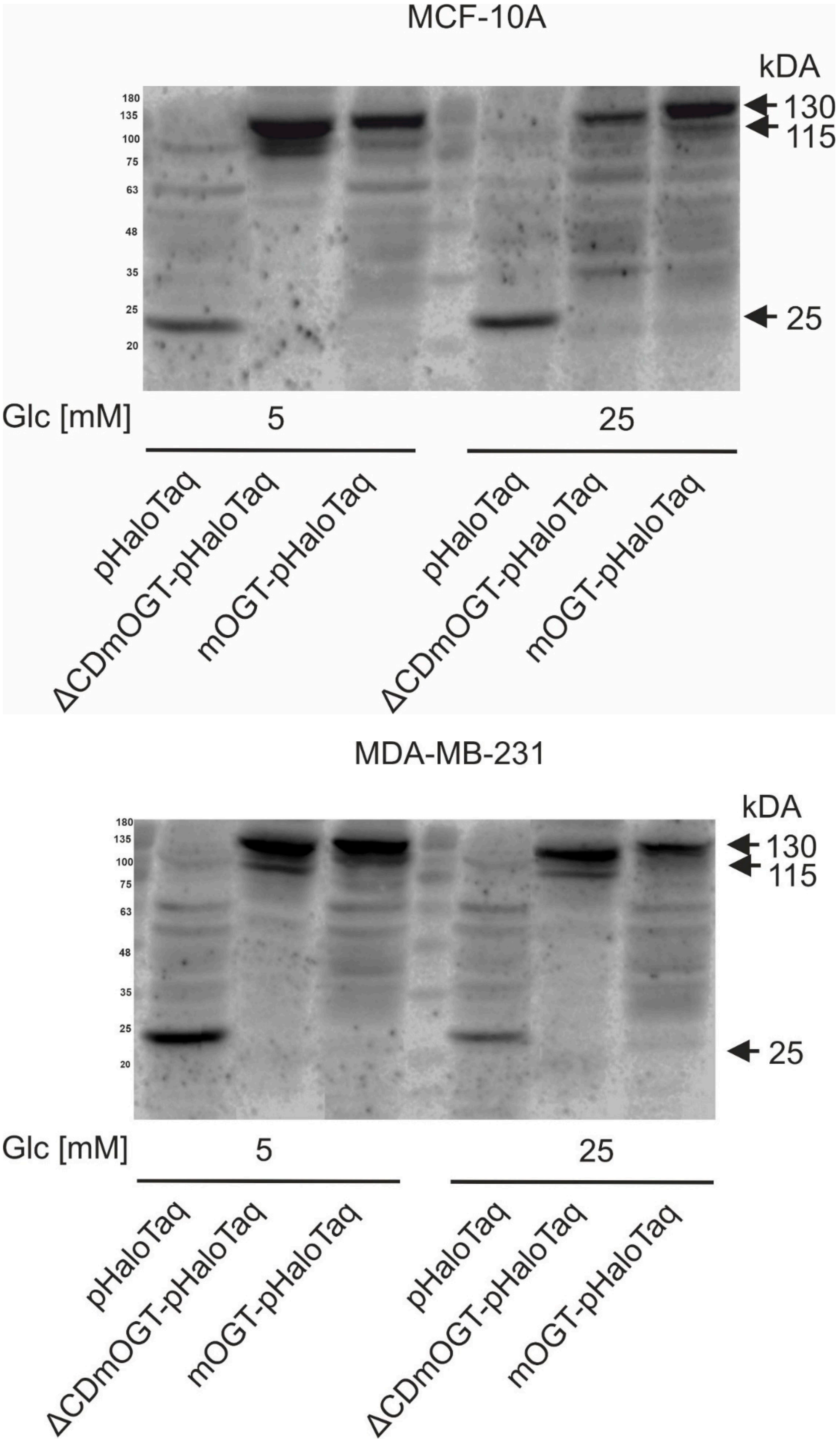
MCF-10A
kDA
180
135
100
75
63
48
35
25
20
130
115
25
Glc [mM]
5
25
pHaloTaq
ΔCDmOGT-pHaloTaq
mOGT-pHaloTaq
pHaloTaq
ΔCDmOGT-pHaloTaq
mOGT-pHaloTaq
MDA-MB-231
kDA
180
135
100
75
63
48
35
25
20
130
115
25
Glc [mM]
5
25
pHaloTaq
ΔCDmOGT-pHaloTaq
mOGT-pHaloTaq
pHaloTaq
ΔCDmOGT-pHaloTaq
mOGT-pHaloTaq

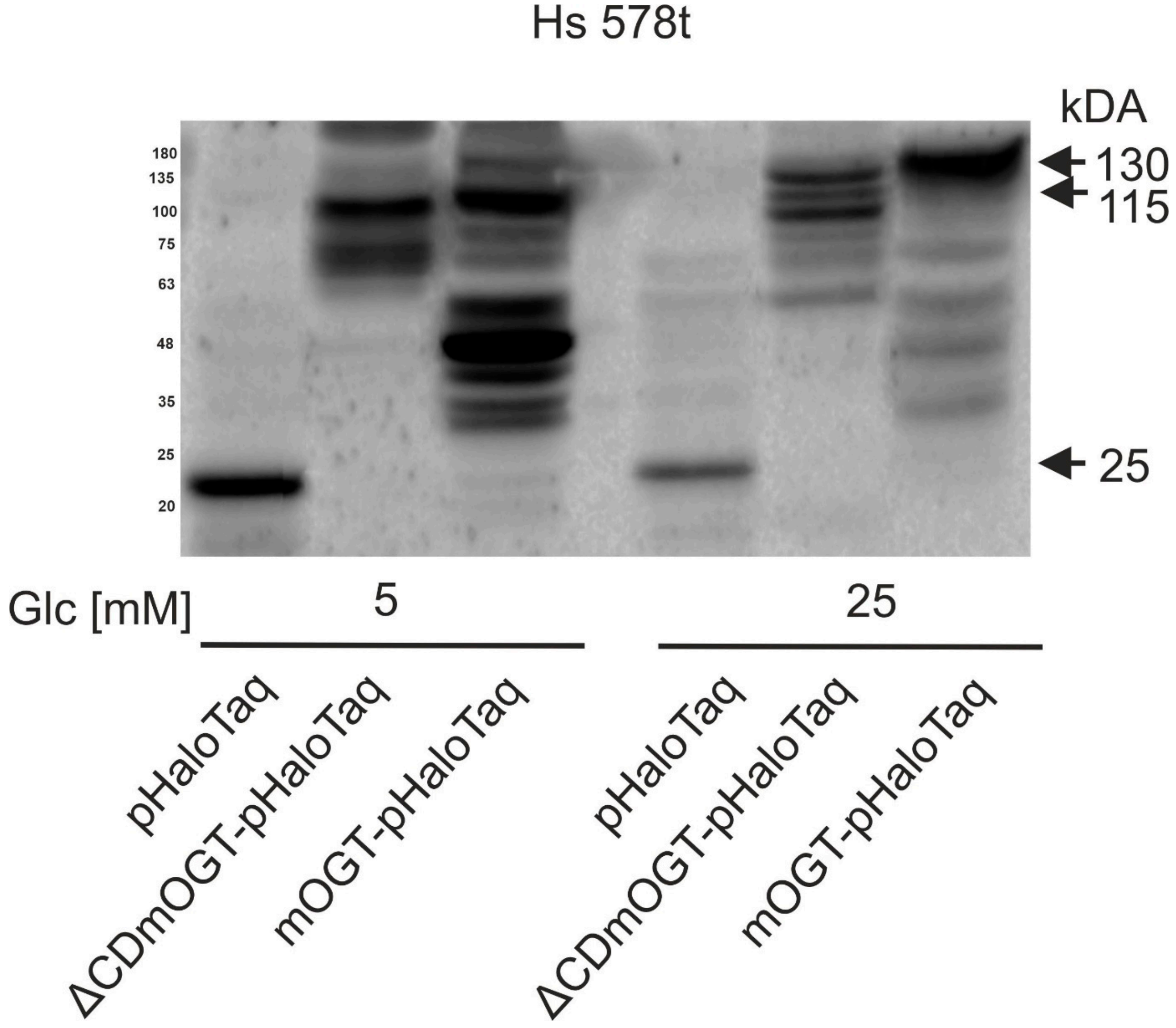
Hs 578t
kDA
180
135
100
75
63
48
35
25
20
130
115
25
Glc [mM]
5
25
pHaloTaq
ΔCDmOGT-pHaloTaq
mOGT-pHaloTaq
pHaloTaq
ΔCDmOGT-pHaloTaq
mOGT-pHaloTaq

Figure 1D

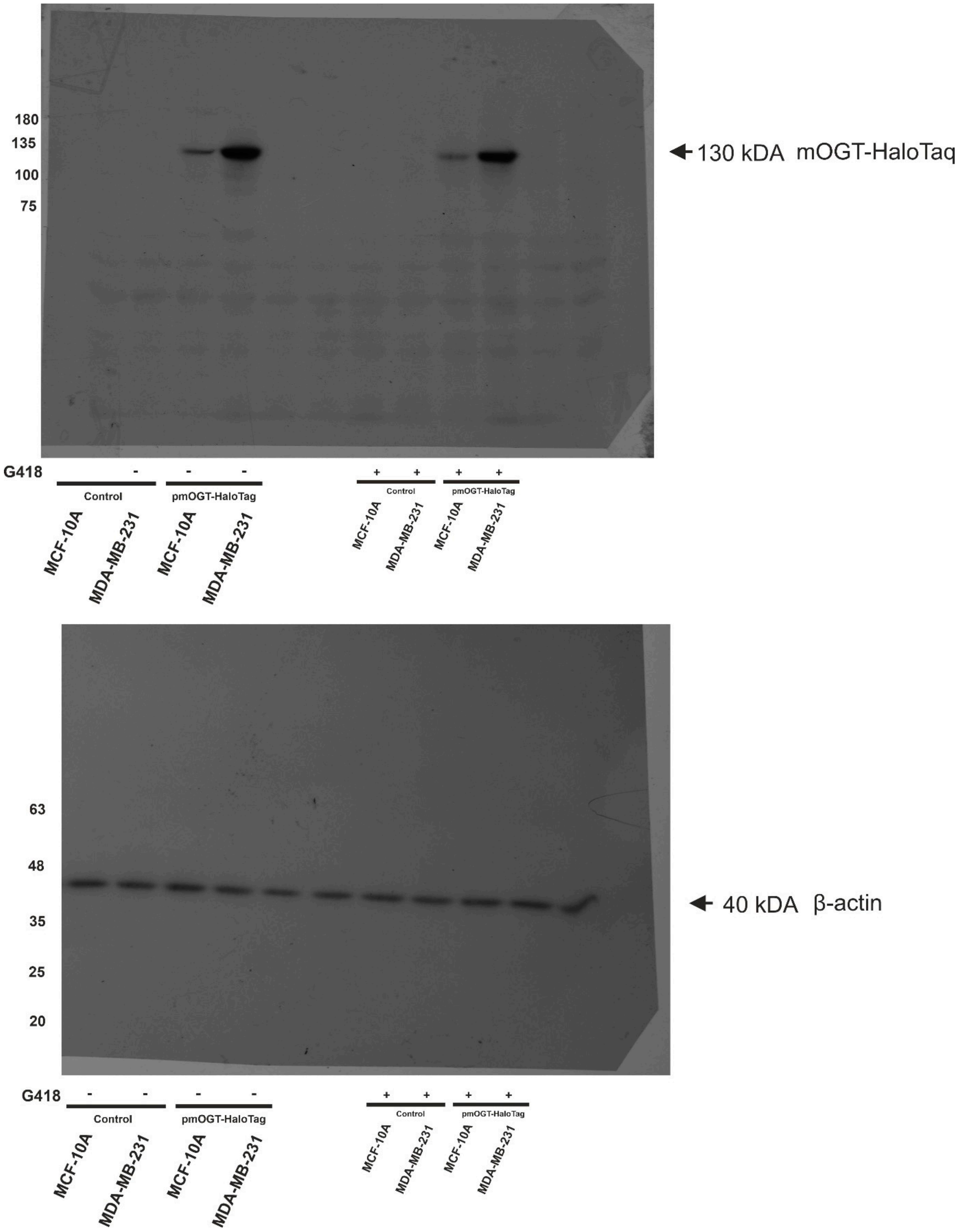
180
135
100
75
130 kDA mOGT-HaloTaq
G418
- - -
+ + + +
Control
pmOGT-HaloTag
MCF-10A
MDA-MB-231
MCF-10A
MDA-MB-231
Control
pmOGT-HaloTag
MCF-10A
MDA-MB-231
MCF-10A
MDA-MB-231
63
48
35
25
20
40 kDA β-actin
G418
- - - -
+ + + +
Control
pmOGT-HaloTag
MCF-10A
MDA-MB-231
MCF-10A
MDA-MB-231
Control
pmOGT-HaloTag
MCF-10A
MDA-MB-231
MCF-10A
MDA-MB-231

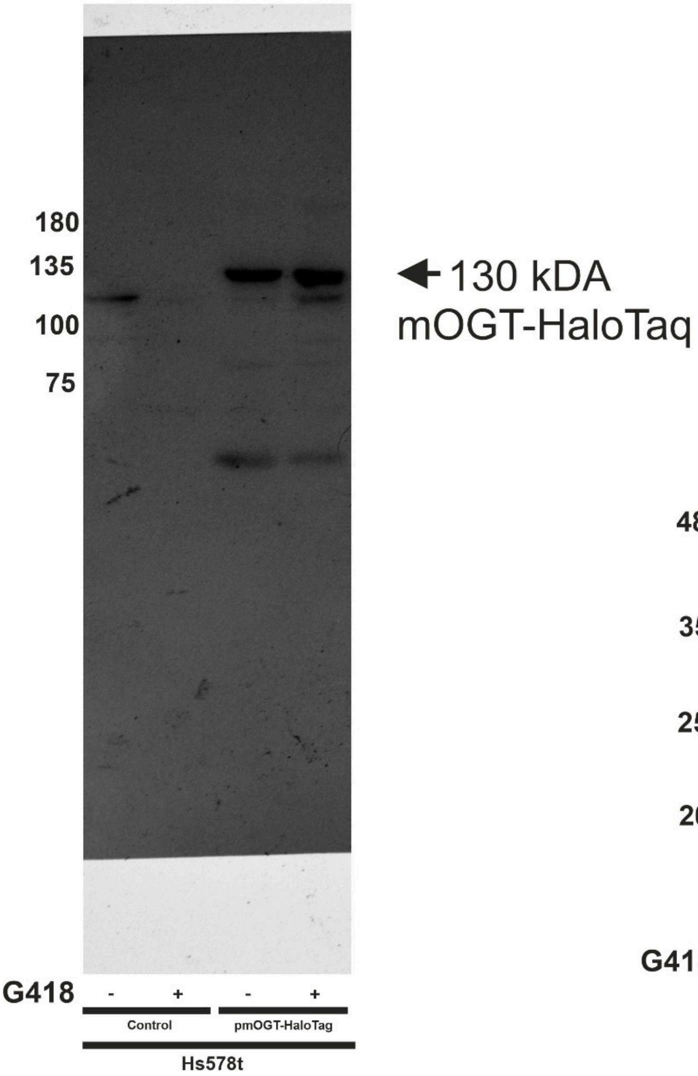
180
135
100
75
130 kDA
mOGT-HaloTaq
G418
-
+
-
+
Control
pmOGT-HaloTag
Hs578t

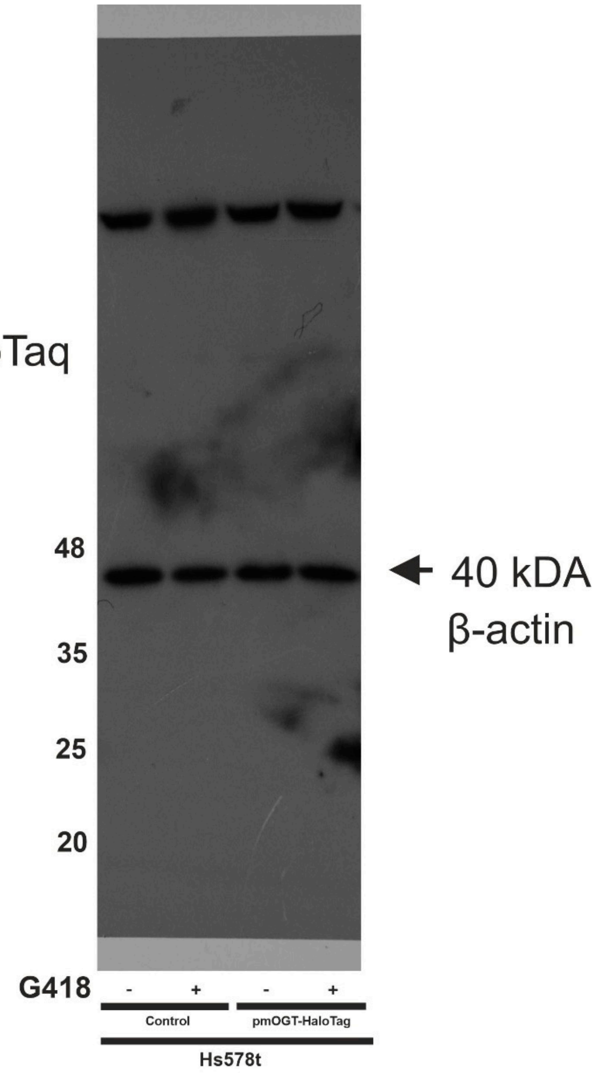
48
35
25
20
40 kDA
β-actin
G418
-
+
-
+
Control
pmOGT-HaloTag
Hs578t

Figure 4A

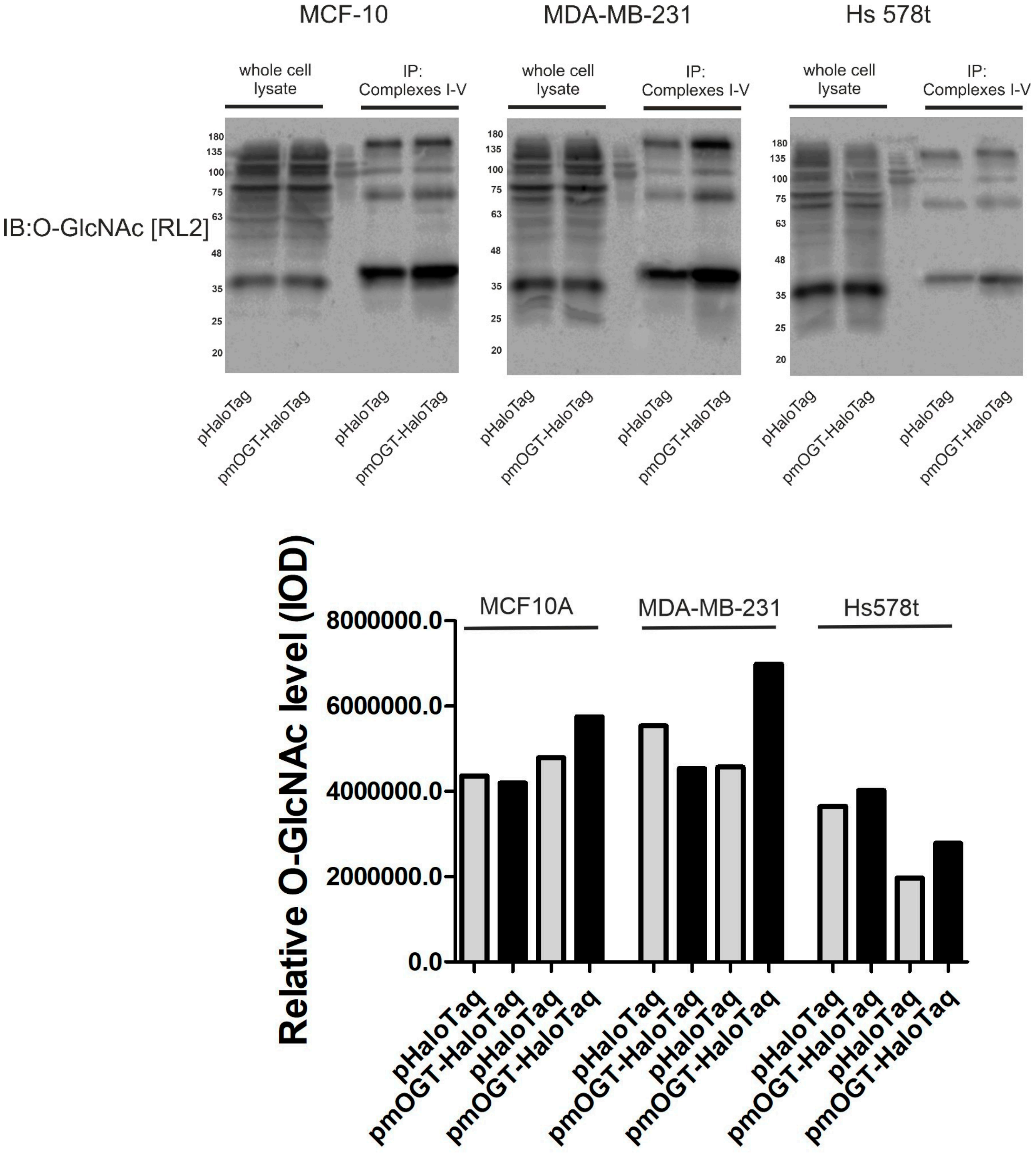
MCF-10
MDA-MB-231
Hs 578t
whole cell lysate
IP: Complexes I-V
IB:O-GlcNAc [RL2]
180
135
100
75
63
48
35
25
20
pHaloTag
pmOGT-HaloTag
Relative O-GlcNAc level (IOD)
MCF10A
MDA-MB-231
Hs578t
8000000.0
6000000.0
4000000.0
2000000.0
0.0
pHaloTaq
pmOGT-HaloTaq

Figure 4B

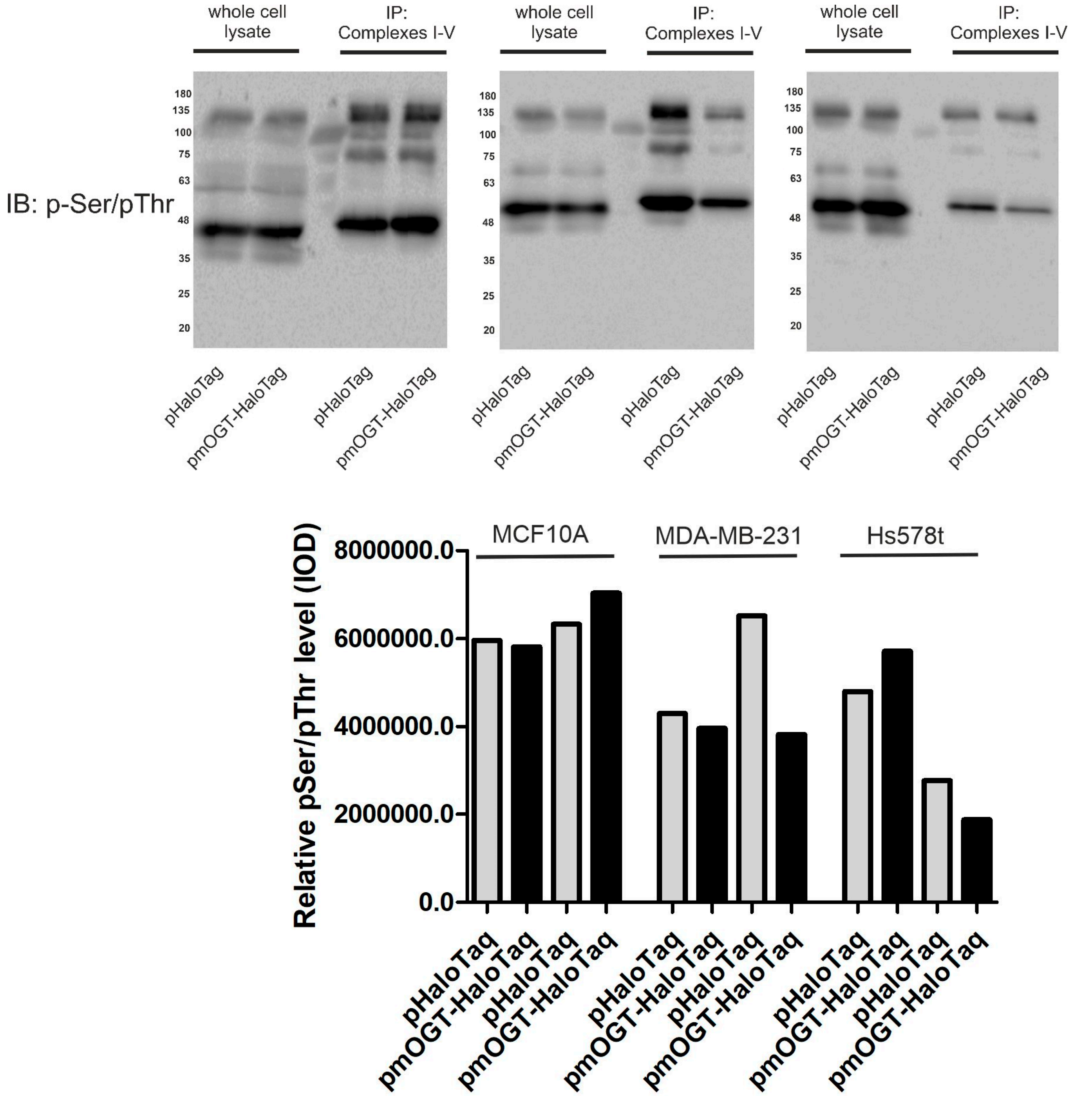
whole cell lysate
IP: Complexes I-V
IB: p-Ser/pThr
180
135
100
75
63
48
35
25
20
pHaloTag
pmOGT-HaloTag
MCF10A
MDA-MB-231
Hs578t
Relative pSer/pThr level (IOD)
8000000.0
6000000.0
4000000.0
2000000.0
0.0
pHaloTaq
pmOGT-HaloTaq

Figure 5Ba

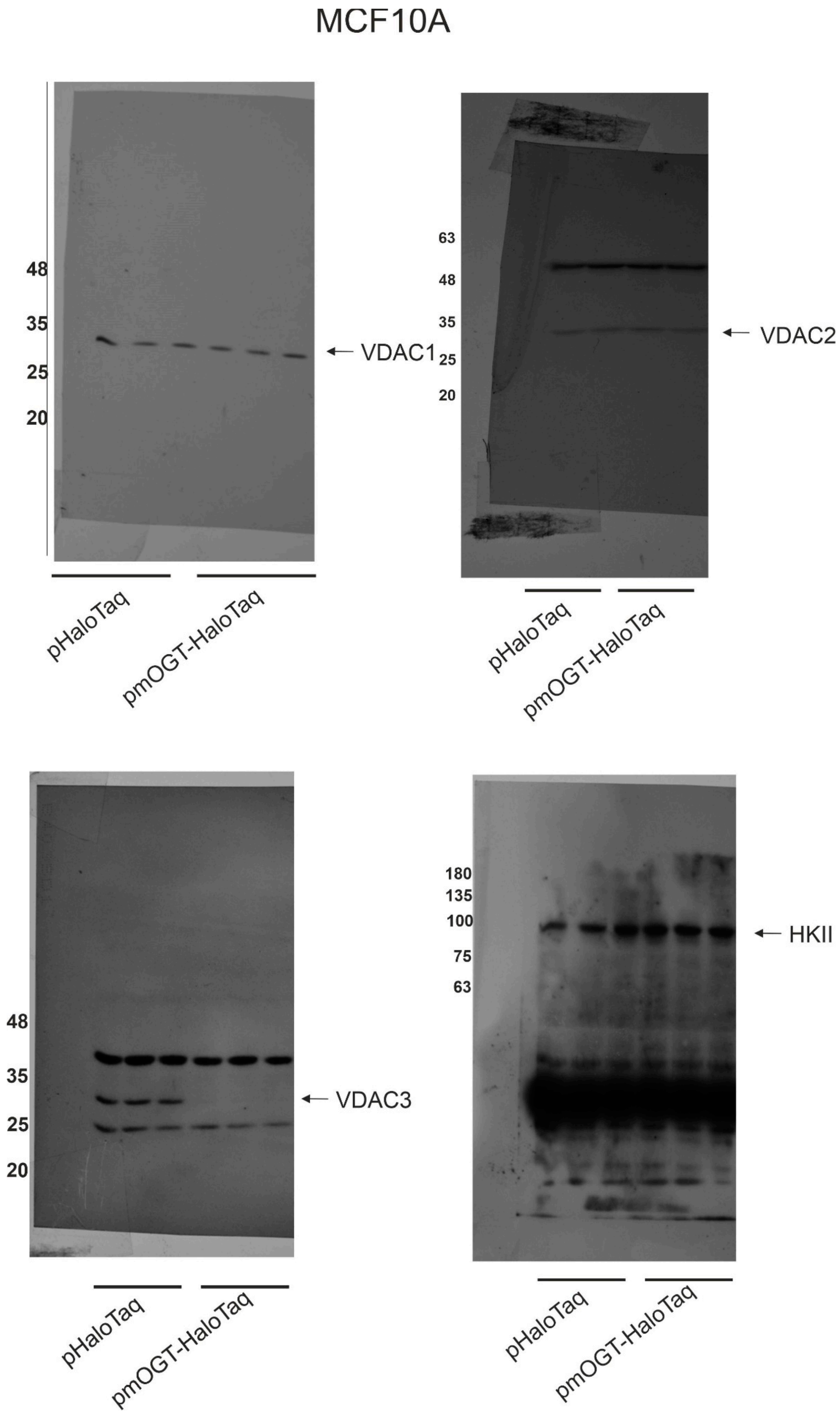
MCF10A
48
35
25
20
VDAC1
pHaloTaq
pmOGT-HaloTaq
63
48
35
25
20
VDAC2
pHaloTaq
pmOGT-HaloTaq
48
35
25
20
VDAC3
pHaloTaq
pmOGT-HaloTaq
180
135
100
75
63
HKII
pHaloTaq
pmOGT-HaloTaq

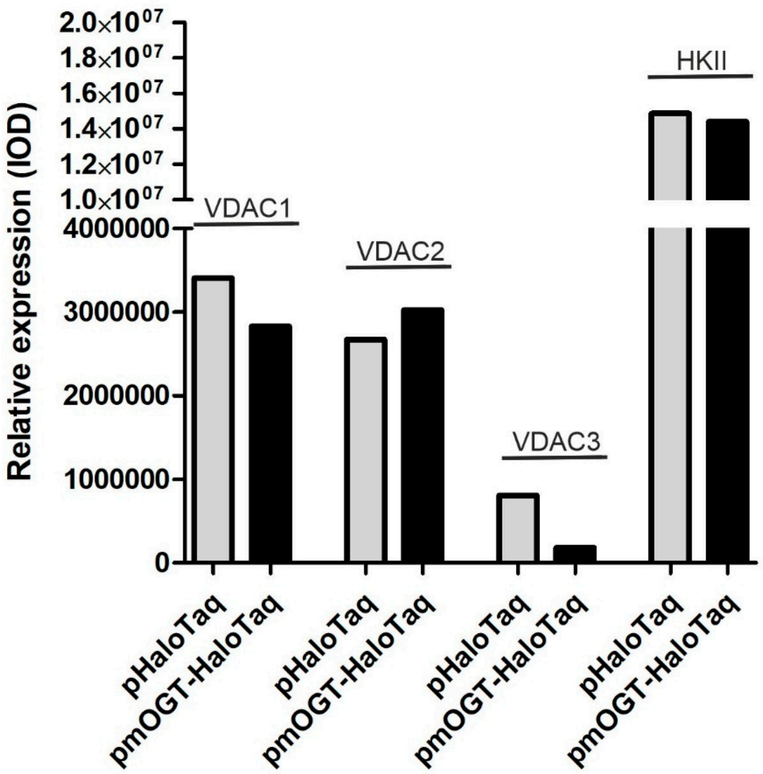

2.0×10^07
1.8×10^07
1.6×10^07
1.4×10^07
1.2×10^07
1.0×10^07
4000000
3000000
2000000
1000000
0
Relative expression (IOD)
VDAC1
VDAC2
VDAC3
HKII
pHaloTaq
pmOGT-HaloTaq
pHaloTaq
pmOGT-HaloTaq
pHaloTaq
pmOGT-HaloTaq
pHaloTaq
pmOGT-HaloTaq

MDA-MB-231

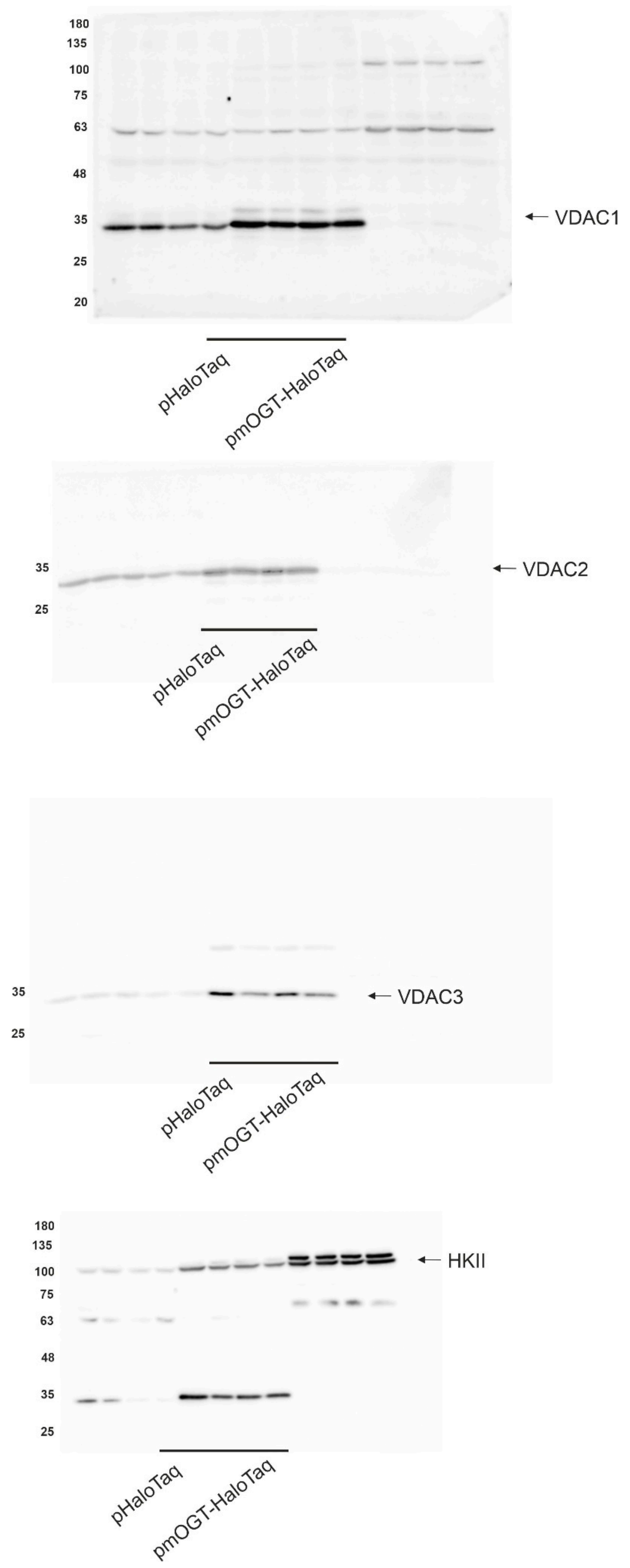
180
135
100
75
63
48
35
25
20
VDAC1
pHaloTaq
pmOGT-HaloTaq
35
25
VDAC2
pHaloTaq
pmOGT-HaloTaq
35
25
VDAC3
pHaloTaq
pmOGT-HaloTaq
180
135
100
75
63
48
35
25
HKII
pHaloTaq
pmOGT-HaloTaq

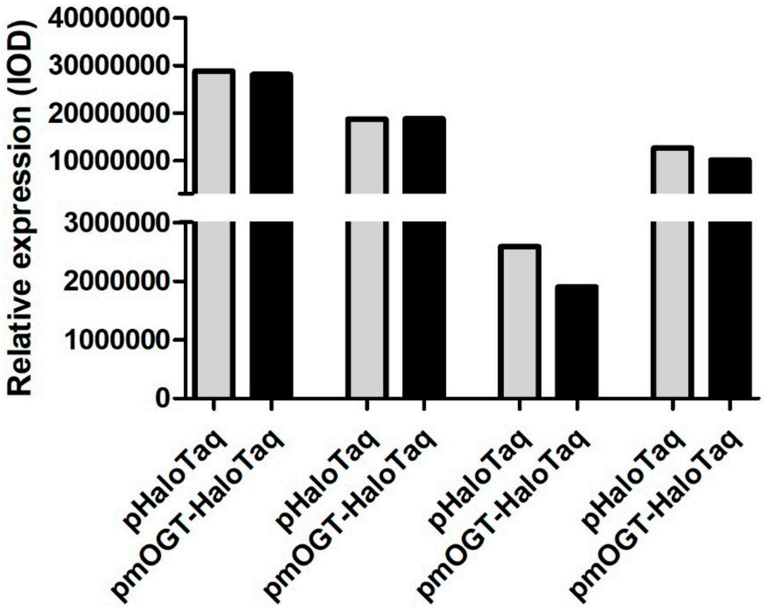

Relative expression (IOD)
40000000
30000000
20000000
10000000
3000000
2000000
1000000
0
pHaloTaq
pmOGT-HaloTaq
pHaloTaq
pmOGT-HaloTaq
pHaloTaq
pmOGT-HaloTaq
pHaloTaq
pmOGT-HaloTaq

Hs578t

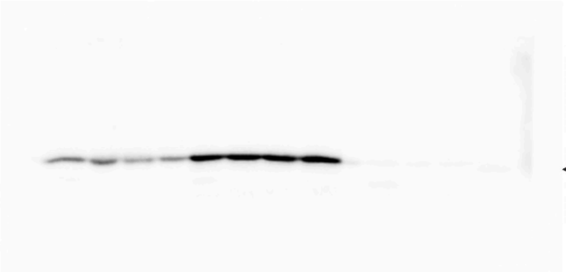

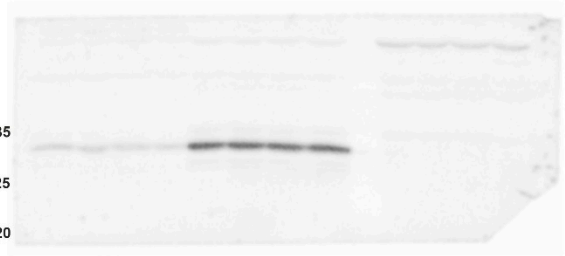

35
25
20
VDAC3
pHaloTaq
pmOGT-HaloTaq

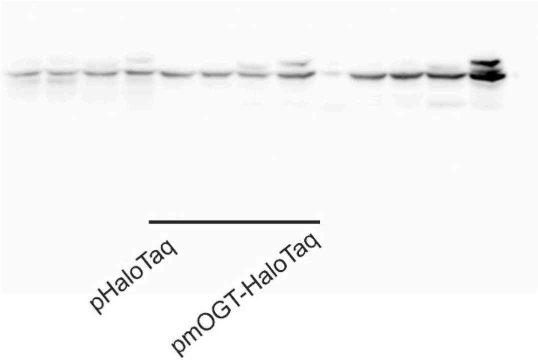

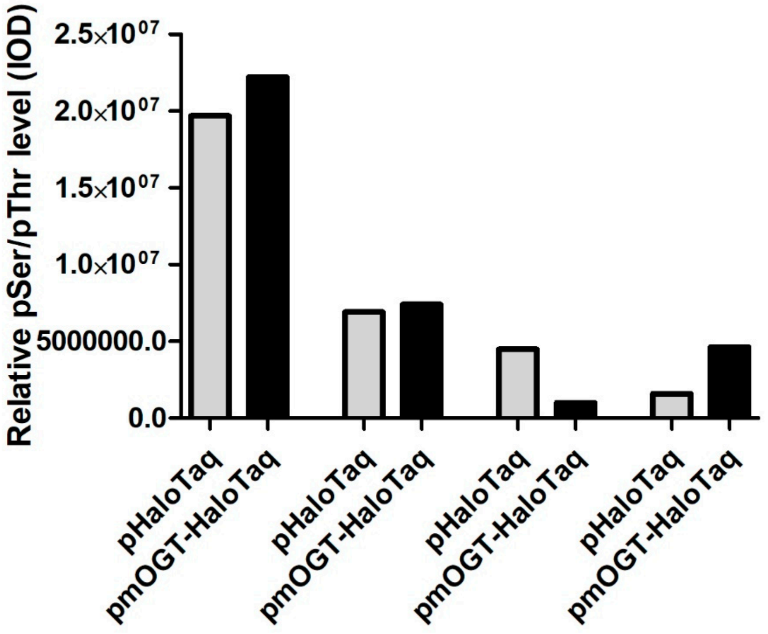
Relative pSer/pThr level (IOD)
2.5×10^07
2.0×10^07
1.5×10^07
1.0×10^07
5000000.0
0.0
pHaloTaq
pmOGT-HaloTaq
pHaloTaq
pmOGT-HaloTaq
pHaloTaq
pmOGT-HaloTaq
pHaloTaq
pmOGT-HaloTaq

Figure 5Bb

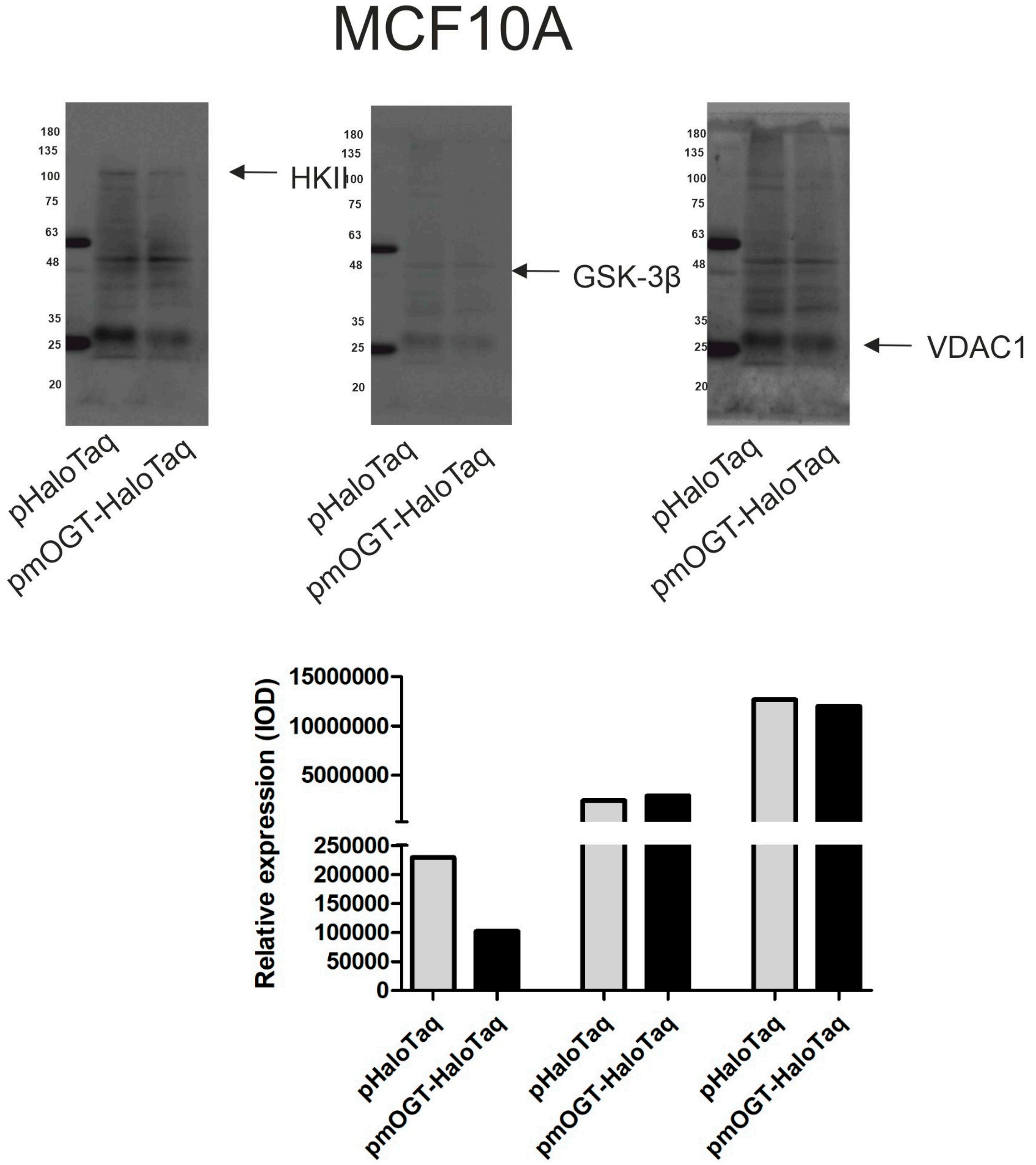
MCF10A
180
135
100
75
63
48
35
25
20
HKII
GSK-3β
VDAC1
pHaloTaq
pmOGT-HaloTaq
Relative expression (IOD)
15000000
10000000
5000000
250000
200000
150000
100000
50000
0

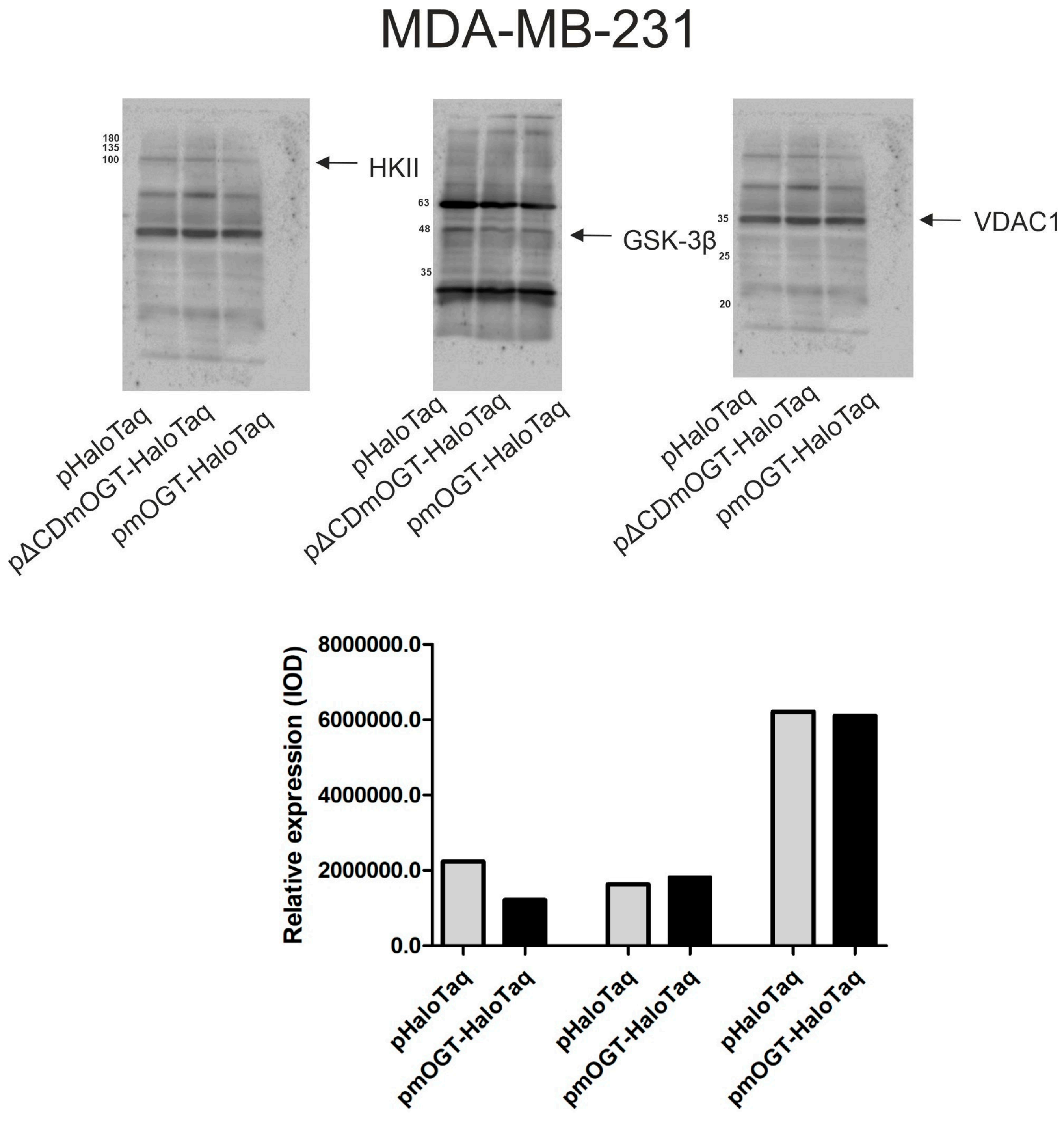
MDA-MB-231
180
135
100
HKII
63
48
35
GSK-3β
35
25
20
VDAC1
pHaloTaq
pΔCDmOGT-HaloTaq
pmOGT-HaloTaq
pHaloTaq
pΔCDmOGT-HaloTaq
pmOGT-HaloTaq
pHaloTaq
pΔCDmOGT-HaloTaq
pmOGT-HaloTaq
Relative expression (IOD)
8000000.0
6000000.0
4000000.0
2000000.0
0.0
pHaloTaq
pmOGT-HaloTaq
pHaloTaq
pmOGT-HaloTaq
pHaloTaq
pmOGT-HaloTaq

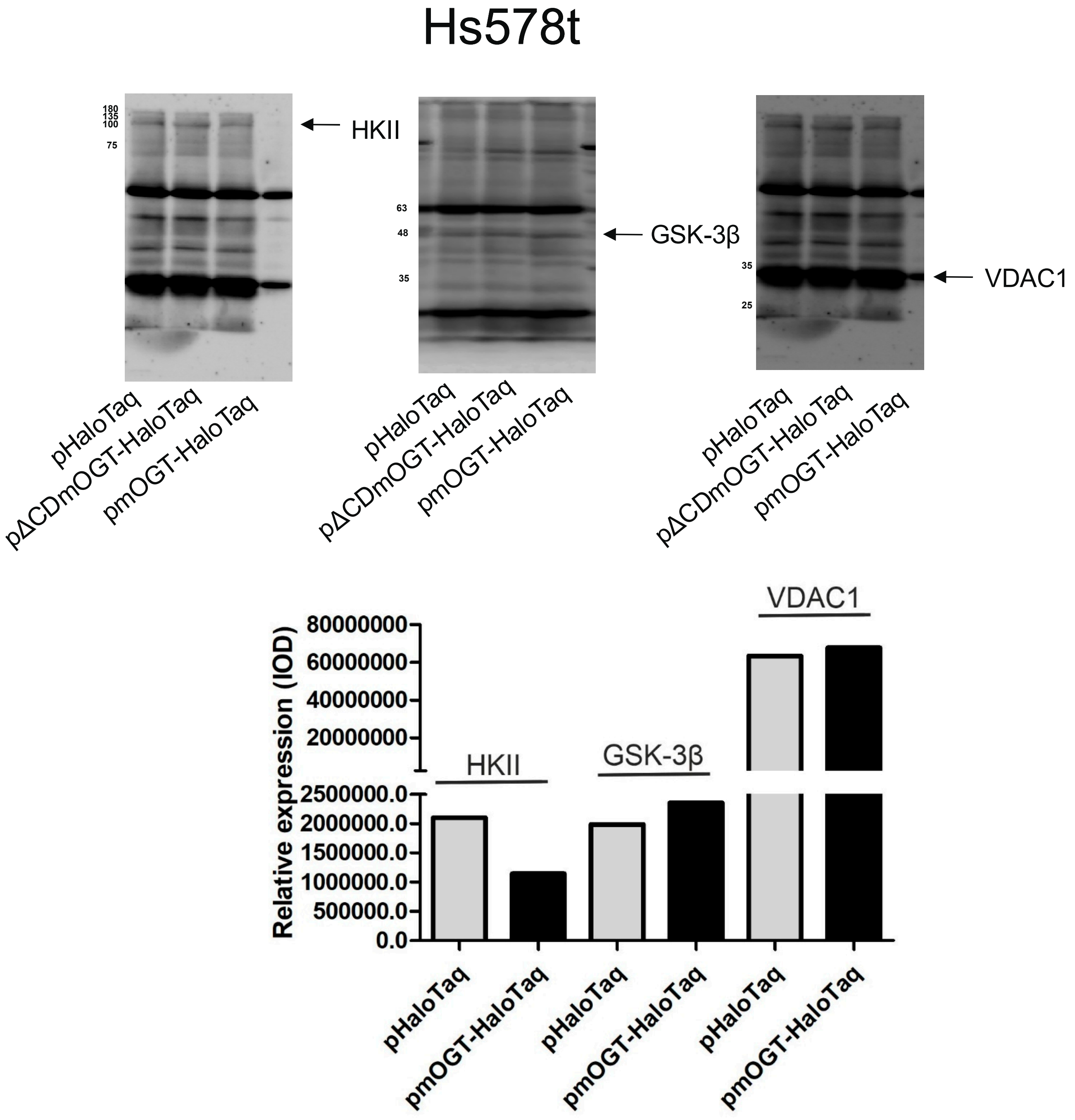

Figure S2. Uncropped original western blot figures.
